# Release kinetics of growth factors loaded into β-TCP ceramics in an *in vitro* model

**DOI:** 10.3389/fbioe.2024.1441547

**Published:** 2024-09-27

**Authors:** Marco Waldmann, Marc Bohner, Anna Baghnavi, Bianca Riedel, Michael Seidenstuecker

**Affiliations:** ^1^ G.E.R.N. Tissue Replacement, Regeneration and Neogenesis, Department of Orthopedics and Trauma Surgery, Medical Center-Albert-Ludwigs-University of Freiburg, Faculty of Medicine, Albert-Ludwigs-University of Freiburg, Freiburg im Breisgau, Germany; ^2^ RMS Foundation, Bettlach, Switzerland

**Keywords:** β-TCP, PRP, ceramic, growth factor, TGF-beta, IGF-1, PDGF-AB

## Abstract

**Introduction:**

β-TCP ceramics are bone replacement materials that have recently been tested as a drug delivery system that can potentially be applied to endogenous substances like growth factors found in blood platelets to facilitate positive attributes.

**Methods:**

In this work, we used flow chamber loading to load β-TCP dowels with blood suspensions of platelet-rich plasma (PRP), platelet-poor plasma (PPP), or buffy coat (BC) character. PRP and BC platelet counts were adjusted to the same level by dilution. Concentrations of TGF-β1, PDGF-AB, and IGF-1 from dowel-surrounding culture medium were subsequently determined using ELISA over 5 days. The influence of alginate was additionally tested to modify the release.

**Results:**

Concentrations of TGF-β1 and PDGF-AB increased and conclusively showed a release from platelets in PRP and BC compared to PPP. The alginate coating reduced the PDGF-AB release but did not reduce TGF-β1 and instead even increased TGF-β1 in the BC samples. IGF-1 concentrations were highest in PPP, suggesting circulating levels rather than platelet release as the driving factor. Alginate samples tended to have lower IGF-1 concentrations, but the difference was not shown to be significant.

**Discussion:**

The release of growth factors from different blood suspensions was successfully demonstrated for β-TCP as a drug delivery system with release patterns that correspond to PRP activation after Ca^2+^-triggered activation. The release pattern was partially modified by alginate coating.

## 1 Introduction

The fundamental idea of drug delivery systems has been around for quite some time and has been tested on many different candidates (Zhang et al., 2011). One of these candidates, β-TCP (Ritschl et al., 2023; Kuehling et al., 2022; Seidenstuecker et al., 2017), could prove beneficial to patients, should the use of this drug delivery system with substances that improve wound healing be successful.

### 1.1 β-TCP

β-TCP ceramics have been in use for over 40 years, starting before 1980, and β-TCP and compound ceramics still play an important role in modern orthopedics and surgery, with hundreds of articles being published annually (Bohner et al., 2020). A variety of positive effects are attributed to their chemical similarity to the mineral phase of bones, including biocompatibility, biodegradability, and osteoinductivity (Humbert et al., 2024; Raymond et al., 2022; Barba et al., 2019; Panseri et al., 2021), which help them remain relevant even after such a long time. In addition to chemical characteristics, a microporous structure was found to further enhance resorption and osteoinduction (Bohner et al., 2020).

As another approach to create even more applications and positive effects, β-TCP ceramics were recently explored as a drug delivery system for clindamycin and vancomycin, which were actively introduced into the ceramics in combination with sodium alginate gel (Ritschl et al., 2023; Kuehling et al., 2022; Seidenstuecker et al., 2017). To the best of our knowledge, no model with a comparable mechanism has yet included any endogenous substances.

The enrichment with growth factors that have been found to accelerate wound healing in animal models and also jawbones of children may have positive effects on β-TCP bone replacement implants that might even exceed the results of other administration methods that have been used in the past, like soaking or simple addition (Szponder et al., 2018; Koksi et al., 2023; Plachokova et al., 2007; Arai et al., 2021; Zhang et al., 2018; Omata et al., 2014).

### 1.2 Growth factors and blood platelets

Growth factors are versatile molecules covering a broad spectrum of functions, including contributions to wound healing.

For example, isoforms of transforming growth factor-beta (TGF-β) were found to influence scar tissue formation. TGF-β1, which is found in adults, leads to increased stimulation of fibroblasts and formation of scar tissue, whereas fetal wounds expressing TGF-β3 instead result in scar-free tissue repair (Ashcroft and Roberts, 2000; Lichtman et al., 2016; Penn et al., 2012). TGF-β has many other functions, including tumor biology and as a master immune regulator (Larson et al., 2020).

Platelet-derived growth factors (PDGFs) are a group of five functional growth factors (Fredriksson et al., 2004) that influence different mesenchymal cells and lead to cell migration of osteoblasts (Kim et al., 2007) and, depending on concentration, migration or proliferation of fibroblasts (De Donatis et al., 2008). They are found in various fibrotic diseases, during wound healing, and take part in embryogenesis (Andrae et al., 2008; Seppä et al., 1982). Surprisingly, recent studies even suggest a potential use of PDGF-AB to improve outcomes after myocardial infarction (Thavapalachandran et al., 2020; Hume et al., 2023).

IGF-1 helps regulate pre- and postnatal growth and angiogenesis and is important for a healthy metabolism. Dysregulation is found in many metabolic diseases (Hakuno and Takahashi, 2018; Shigematsu et al., 1999).

In muscle and tendon injuries, IGF-1 can also reduce swelling and modulate inflammation, with one mechanism being the upregulation of M2 macrophages, which are known to reduce inflammation and express additional growth factors, including TGF-β1, PDGF, and VEGF (Wynn et al., 2013; Tonkin et al., 2015; Prabhath et al., 2018). Effects between IGF-1 and other growth factors can also positively influence ligament and tendon healing (Prabhath et al., 2018; Tsuzaki et al., 2000; Hagerty et al., 2012).

PDGFs, TGF-β, and IGF-1 can all be found in blood platelets (Karey and Sirbasku, 1989; Chan and Spencer, 1998; Blair and Flaumenhaft, 2009; Assoian et al., 1983) and consequently in increased concentrations in platelet-rich plasma (PRP) (El-Sharkawy et al., 2007). PRP has been produced by many different methods with varying outcomes, but generally speaking, it results in a blood suspension rich in platelets. The accumulated platelets can be activated in various ways, such as by contact with Ca^2+^ and collagen, as found during wound healing, or by cell membrane lysis triggered by freeze-thaw cycles (Durante et al., 2013; Semple et al., 2011).

In this study, we examine the release patterns of growth factors from microporous β-TCP ceramics previously loaded with PRP compared to other blood suspensions (buffy coat, platelet-poor plasma) using flow chamber loading as a potential new delivery system for growth factors. We also address the influence of sodium alginate coating of said ceramics on the release pattern.

## 2 Material and methods

**Table:** 

Material	Information
Ethanol 99%	SAV Liquid Production GmBH, Flintsbach am Inn, Germany
Casy Cell Counter and Analyzer Model TT	OMNI Life Science, Bremen, Germany
60 μm capillary for Casy TT	OMNI Life Science, Bremen, Germany
Gibco DMEM, high glucose, no glutamine, no calcium	Thermo Fisher, Waltham, United States, 21068028
Alginic acid sodium salt from brown algae (medium viscosity)	Sigma-Aldrich, Darmstadt, Germany, A2033
NaCl 0.9% solution	B. Braun Melsungen, Melsungen, Germany, 3570160
Quantikine ELISA Human PDGF-AB Immunoassay	Sigma-Aldrich, Darmstadt, Germany, DHD00C
Quantikine ELISA Human IGF-I/IGF-1 Immunoassay	Sigma-Aldrich, Darmstadt, Germany, DG100B
Quantikine ELISA Human TGF-β1 Immunoassay	Sigma-Aldrich, Darmstadt, Germany, DB100C
Memmert UN110	Memmert GmbH & Co. KG, Schwabach, Germany
SpectroStar Nano	BMG Labtech, Ortenberg, Germany

### 2.1 β-TCP

To create β-TCP dowels with our specifications, the Robert Mathys Foundation (RMS) mixed 80 g of a-tricalcium phosphate and 20 g of tricalcium phosphate (Art. No. 102143, Merck; a mixture of apatite and some calcium hydrogenphosphate) with 60.0 ± 0.2 g of a solution containing 0.2M Na_2_HPO_4_ and 1% polyacrylic acid (Art. No. 81132, Fluka; Mw = 5.1 kDa). It was stirred (2.5 min), and the paste was poured into plastic syringes with the tips removed (Ø = 23 mm, 70 mm length). The paste hardened for 45 min, then was covered with 10 mL of PBS (pH 7.4) solution (Art. No. P5368, Sigma) and incubated for 3 days at 60°C. The samples (Ø = 23 mm; L = 70 mm) were dried at the same temperature and sintered at 1,250°C for 4 h. Heating and cooling took place at 1°C/min. The cylinders were trimmed to a length of 25 mm and a diameter of 7 mm. At the end, the samples were washed in ethanol to remove residual particles and calcined at 900°C to burn off all organic residues. Before use, the ceramics were shortened to cylinders with a length of 6 mm and washed again. The samples were dry sterilized at 200°C for 4 h (Memmert UN110, drying oven) (Stahli et al., 2010).

An analysis using environmental scanning electron microscopy (ESEM) showed a mean pore diameter of 4.8 ± 1.2 µm or an average pore radius of 2.1 ± 0.3 µm for the β-TCP ceramics produced with this method (Seidenstuecker et al., 2017; Seidenstuecker et al., 2021).

After the initial production, the β-TCP dowels provided by the RMS were cut into 6-mm-thick slices. To eliminate potential impurities introduced during this process, they afterward were first washed in an ultrasonic bath, starting with 70% ethanol and followed by distilled water for 10 min each. After subsequent autoclaving, the slices were ready for flow chamber loading (Waldmann et al., 2024).

### 2.2 Blood sampling

Blood products were provided by the Institute for Transfusion Medicine and Gene Therapy, University Freiburg.

A 30 mL sample of EDTA whole blood was pooled and further processed into either platelet-rich plasma (PRP) or platelet-poor plasma (PPP) by centrifugation. PRP, in accordance with Cattaneo et al. (2013), was centrifuged for 10 min at 200 g without breaks, and the supernatant of the erythrocyte fraction was transferred into a new vessel.

PPP, intended as a control with mostly circulating levels of growth factors, was created by centrifuging for 5 min at 900 g, followed by a transfer and subsequent centrifugation at 1,500 g for 15 min of the supernatant of the erythrocyte fraction. The upper 2/3 of the resulting supernatant was transferred again to receive PPP (Cattaneo et al., 2013).

Buffy coat (BC) were used without further centrifugation steps.

Platelet counts were determined using a CASY TT cell counter according to the manufacturer’s instructions.

BC and PRP were diluted with saline solution (Supplementary Material 1), according to the determined platelet count results, to receive uniform counts of 450,000 platelets/μL. PPP was accepted with counts below 100,000 platelets/μL and did not require dilution.

### 2.3 Loading using flow chambers

Loading via flow chambers made of stainless steel was performed as previously described (Waldmann et al., 2024; Seidenstuecker et al., 2015).

For each loading process, a 6 mm β-TCP slice was placed inside a silicone seal and enclosed within a flow chamber. A vacuum of 350 mbar was used to load 1 mL of sample material (BC, PRP, or PPP). The process was stopped after 30 s, and the chamber reopened to retrieve the silicone seal holding the ceramic.

After breaking the silicone seal, 6 mm slices were blotted against paper towels to remove excess fluid. Afterward, each 6 mm slice was transferred into a separate well of a 24-well plate holding 2 mL of calcium-free DMEM.

A second group was created by placing some slices into 1% alginate for 30 s before transferring them into calcium-free DMEM. No crosslinking using Ca^2+^ was performed. A total of n = 3 for plasma suspensions of each type (PRP, PPP, BC) and either alginate or no alginate treatment were examined. The same original plasma suspensions were used for loading in groups with and without alginate treatment.

### 2.4 Sample collection

The 24-well plates were incubated at 37°C for up to 5 days. Samples were taken at 24-h intervals starting 2 h after loading, resulting in analysis times of 2 h, 26 h, 50 h, 74 h, and 98 h after loading. Samples were taken by pipetting 180 µL of culture medium into an Eppendorf tube that was immediately placed on ice. The removed 180 µL were replaced with the appropriate medium during each sampling.

### 2.5 Storage

The samples taken were centrifuged at 7,500 g for 5 min (Durante et al., 2013), and the resulting platelet-free supernatants were stored as aliquots at −80°C.

### 2.6 ELISA

Prepared aliquots were analyzed using ELISA-Kits from R&D Systems following the manufacturer’s instructions for assays of serum samples for the growth factors TGF-β1, PDGF-AB, and IGF-1. Concentrations within these assays were determined using a SpectroStar Nano spectrometer.

### 2.7 Statistics

All data shown are presented as mean ±95% confidence interval. One-way analysis of variance ANOVA was performed for all groups at each time interval, followed by multiple comparisons with Fisher’s exact test at a significance level of 0.05. Significantly different results according to Fisher’s least significant difference (LSD) test are highlighted within the figures. Origin 2021 Professional SR1 (OriginLab, Northampton, MA, United States) was used for statistical calculations.

## 3 Results

We analyzed the concentrations of growth factors released from plasma suspension-loaded dowels into the surrounding medium by means of ELISA. Hereinafter, the results are presented for each individual growth factor (TGF-β1, PDGF-AB, IGF-1) regarding different time points after loading as well as different plasma suspensions.

### 3.1 TGF-β1

**FIGURE 1:**
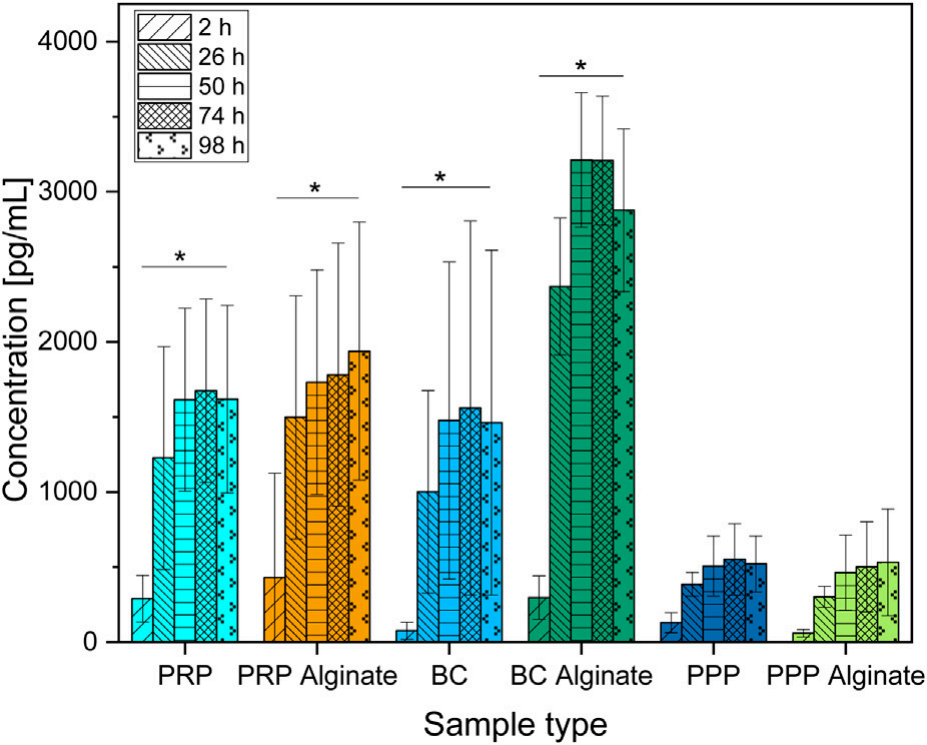
Absolute concentration of TGF-β1 organized in groups for each sample type showing different time points progressing from left to right. Significant results between different time points for the same sample are presented within the picture according to Fisher’s exact test with a significance level of 0.05. The significance between different groups at the same time is shown in Figure 2.

**FIGURE 2:**
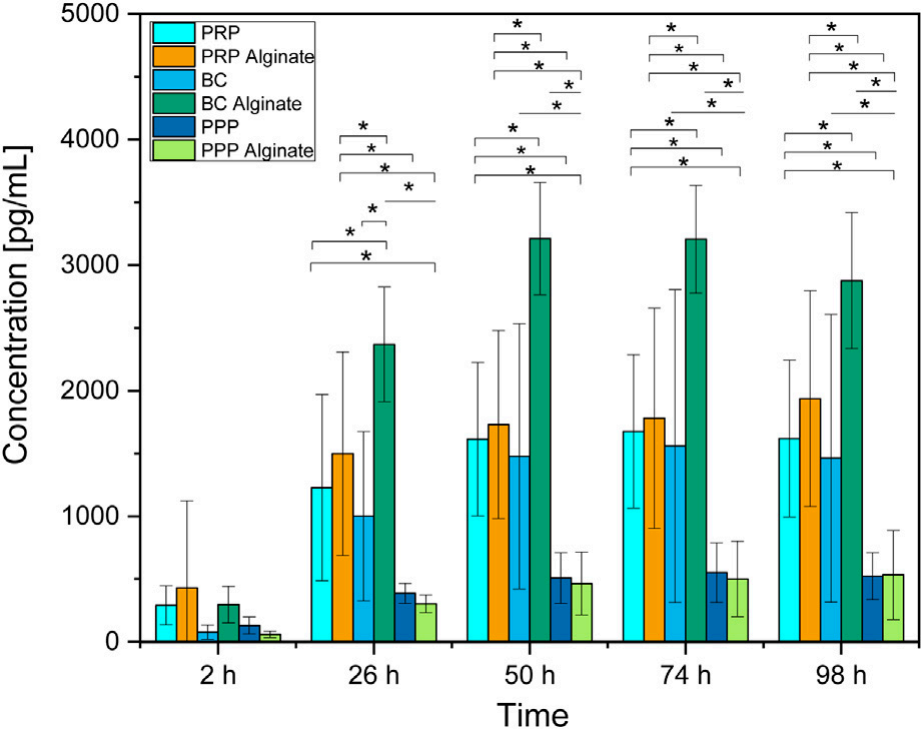
Absolute concentration of TGF-β1 for each analyzed time point compared between all sample types analyzed. Significant results between different groups at the same time are presented within the picture according to Fisher’s exact test with a significance level of 0.05. The significance between different time points for the same sample type is shown in Figure 1.

Slices loaded with PRP, PRP alginate, BC, and BC alginate showed a significant increase of TGF-β1 compared between 2 h after loading and all later time points analyzed. PPP and PPP alginate showed no significant increase (Figure 1). No sample type had a significantly higher concentration 2 h after loading than any other group. PRP and BC showed higher concentrations than PPP, starting at 50 h after loading. For all times after 2 h, BC alginate had higher concentrations than PRP alginate and PPP alginate. PRP alginate also showed higher concentrations than PPP alginate for all times except for 2 h after loading (Figure 2).

Groups additionally treated with alginate showed higher concentrations than their untreated counterparts only for BC alginate compared to BC for all time points except 2 h after loading. No significant differences were found between PRP and PRP alginate or between PPP and PPP alginate (Figure 2).

### 3.2 PDGF-AB

**FIGURE 3:**
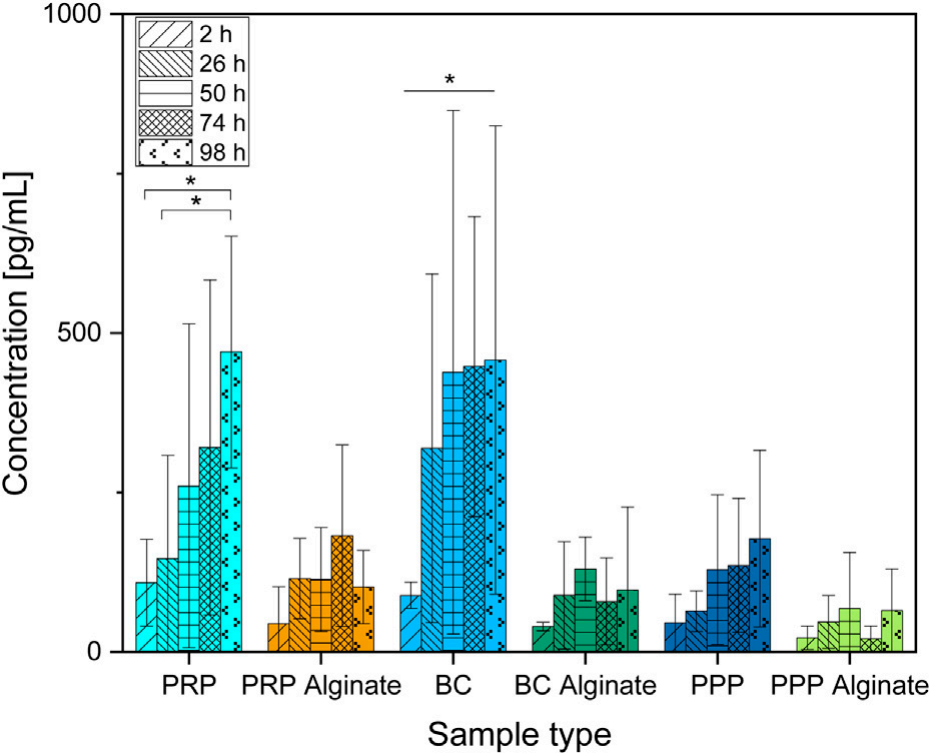
Absolute concentration of PDGF-AB organized in groups for each sample type showing different time points progressing from left to right. Significant results between different time points for the same sample are presented within the picture according to Fisher’s exact test with a significance level of 0.05. The significance between different groups at the same time is shown in Figure 4.

Only slices loaded with BC showed a significant increase of PDG-AB compared between 2 h after loading and all later time points analyzed. PRP slices showed increased concentrations after 98 h compared to 2 h and 26 h after loading. PRP alginate, BC alginate, PPP, and PPP alginate showed no significant increase (Figure 3).

**FIGURE 4:**
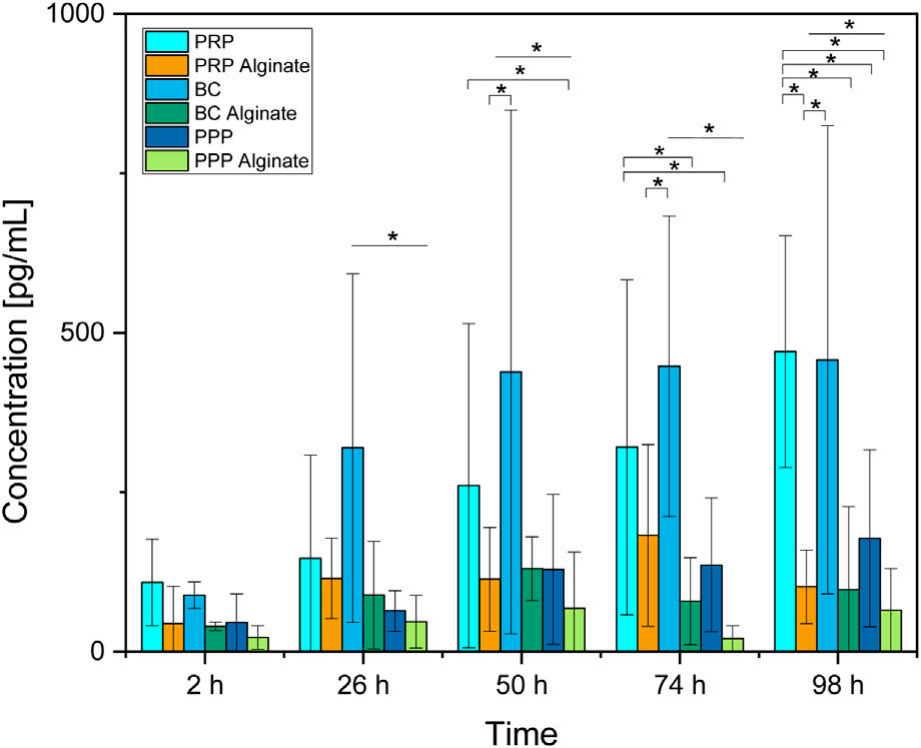
Absolute concentration of PDGF-AB for each analyzed time point compared between all sample types analyzed. Significant results between different groups at the same time are presented within the picture according to Fisher’s exact test with a significance level of 0.05. The significance between different time points for the same sample type is shown in Figure 3.

Between groups, the concentration for BC was higher than the concentration for PPP at all time points except 2 h. The concentration for PRP was only higher than that for PPP after 98 h (Figure 4).

No significant difference between the three alginate groups was found at any time. BC had higher concentrations than BC alginate at every point in time except for 2 h after loading. PRP had higher concentrations than PRP alginate at 98 h after loading. PPP and PPP alginate did not show significantly different concentrations (Figure 4).

### 3.3 IGF-1

**FIGURE 5:**
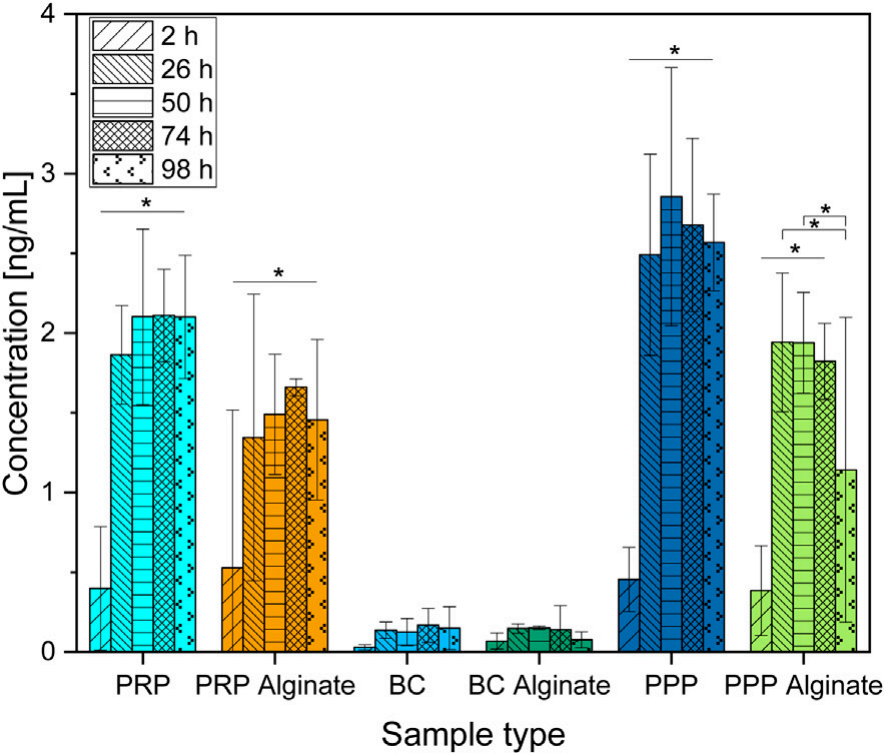
Absolute concentration of IGF-1 organized in groups for each sample type showing different time points progressing from left to right. Significant results between different time points for the same sample are presented within the picture according to Fisher’s exact test with a significance level of 0.05. The significance between different groups at the same time is shown in Figure 6.

The IGF-1 concentration compared to 2 h after loading increased for all time points for PRP, PRP alginate, and PPP. Concentrations for PPP alginate increased for 26 h, 50 h, and 74 h compared to 2 h after loading and were lower at 26 h and 50 h than at 98 h after loading (Figure 5).

**FIGURE 6:**
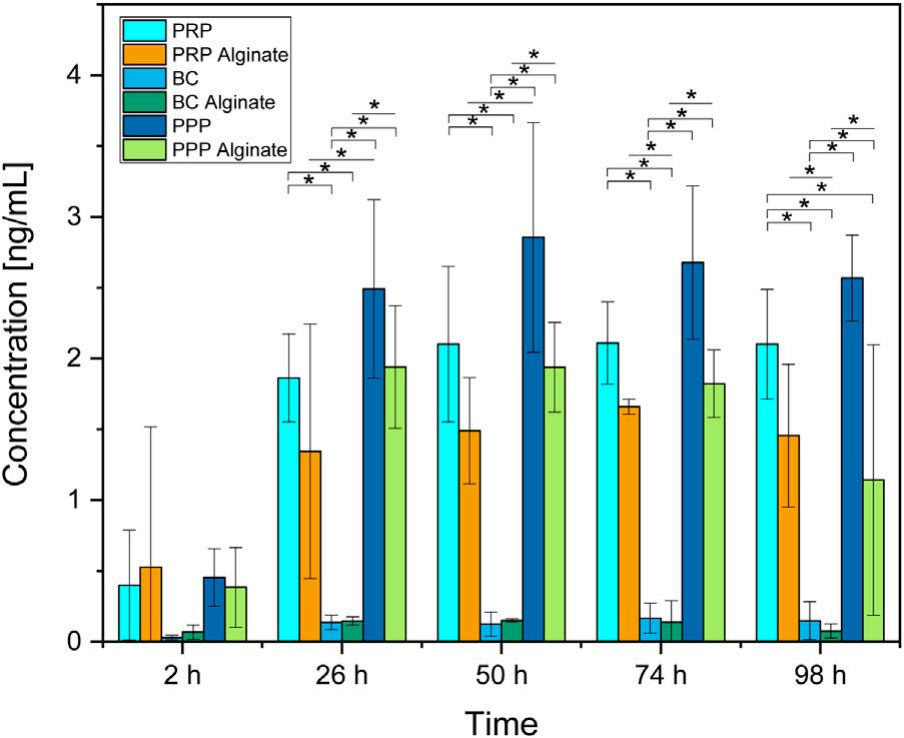
Absolute concentration of IGF-1 for each analyzed time point compared between all sample types analyzed. Significant results between different groups at the same time are presented within the picture according to Fisher’s exact test with a significance level of 0.05. The significance between different time points for the same sample type is shown in Figure 5.

PPP and PRP had higher concentrations than BC at every time point, including 2 h after loading. Concentrations between PRP and PPP did not differ significantly (Figure 6).

Concentrations found for PRP alginate and PPP alginate were higher than for BC alginate at all time points except for 2 h after loading. PRP alginate and PPP alginate did not have significantly different concentrations. Groups additionally treated with alginate showed no significant differences compared to their untreated counterparts but tended to have lower concentrations (Figure 6).

## 4 Discussion

Although no difference was detectable between groups 2 h after loading, results for TGF-β1 concentrations increased for every later point in time, with results for PRP, PRP alginate, and BC being equally high. Results for BC alginate achieved higher concentrations than all other groups, while PPP and PPP alginate, as control groups, had the lowest concentrations (Figure 2).

Concentrations for PRP, PRP alginate, BC, and BC alginate increased significantly between 2 h and 26 h but not thereafter. A similar increase pattern was observed for PPP and PPP alginate (Figure 1), and the difference was not significant.

With each individual test group exceeding our controls, a release of TGF-β1 by platelets and a subsequent diffusion from within the ceramic into the culture medium seems certain. Surprisingly, alginate treatment did not lead to delayed or at least unaffected release times but rather led to higher concentrations for BC alginate than BC.

Concentrations of PDGF-AB increased significantly over time for PRP and BC, while concentrations of their alginate counterparts and PPP/PPP alginate controls did not increase (Figure 3). Concentrations found in PRP samples became significantly higher than for PRP alginate and PPP after 98 h, while BC samples had higher concentrations than BC alginate and PPP starting after 26 h. PRP alginate, BC alginate, PPP, and PPP alginate never showed significant increases and stayed at equally low concentration levels (Figure 4).

Because both PRP and BC increased over time, and PPP controls had low concentrations, a release of growth factors from platelets, as already seen for TGF-β1, into the surrounding medium seems likely. The missing increase found in alginate groups indicates that alginate coating prevents the release of PDGF-AB into the surrounding medium for at least 98 h.

The faster release from BC than PRP might be due to a higher dilution of BC. Pores (⌀ 4.8 ± 1.2 µm) of the ceramic could potentially be obstructed by remaining blood cells and slow the release of PDGF-AB into the surrounding medium. Dissolution of β-TCP as well as the reaction of alkaline phosphatase, which can be found in blood, with β-TCP releases Ca^2+^ (Egorov et al., 2022; Gomori and Benditt, 1953; Epple, 2003) and could, therefore, also reactivate calcium-dependent blood coagulation proteins as another possible explanation for blocked pores. The necessary dilutions to receive uniform platelet counts between PRP and BC samples were higher for BC (Supplementary Material 1). Although concentration differences of platelets were hereby largely reduced, the remaining blood components such as different cells, coagulation proteins, or alkaline phosphatase could, therefore, be present in much lower concentrations in (diluted) BC and thus result in less obstruction, respectively achieving faster release but ultimately comparable PDGF-AB concentrations between PRP and BC.

Results for IGF-1 differed greatly compared to the other two growth factors analyzed. Concentrations of PRP, PRP alginate, PPP, and PPP alginate controls increased over time, while BC and BC alginate concentrations remained low (Figure 6). BC had significantly lower concentrations than the PRP and PPP groups starting at 26 h. The PRP and PPP groups never differed significantly, but PRP had slightly lower results. The alginate groups for BC and PRP were not significantly lower than groups without alginate but tended to be lower at every point of time except 2 h, making a slight decrease in IGF-1 release due to an alginate coating likely (Figure 5).

With the highest concentrations found in PPP and BC not increasing at all, the increase cannot be attributed to release from platelets. In addition to platelets (Karey and Sirbasku, 1989; Chan and Spencer, 1998), IGF-1 is also found at relevant circulating levels (Murphy et al., 2020; Castro-Diehl et al., 2021; Peila and Rohan, 2024). Assuming circulating concentrations as the main component and taking the different levels of dilution into account (Supplementary Material 1), the results of PPP, without dilution, slightly exceeding PRP, with low dilution, and strongly exceeding BC, with high dilution, can very well be explained by a missing or low release from platelets and circulating levels as the driving force.

The abovementioned release of Ca^2+^ from β-TCP (Egorov et al., 2022; Gomori and Benditt, 1953; Epple, 2003) also provides a rational explanation for the release pattern found in this work. An increased release of TGF-β and PDGF-AB from platelets taking place over 30 min without IGF-1 elevation was previously demonstrated by Durante et al. (2013) after the direct addition of CaCl_2_ to PRP. Considering that the reaction of alkaline phosphatase and β-TCP needs time to release Ca^2+^, calcium ions as a main trigger for the observed growth factor increases, which mainly happened between 2 h and 26 h after loading, seem very likely.

Diffusion from hydrogels such as alginate is generally reduced for larger molecules (Caballero Aguilar et al., 2018; Caballero Aguilar et al., 2019). The release inhibition found for PDGF-AB from alginate-treated samples thus might depend on molecular size. With 28–31 kDA (Hammacher et al., 1988; Antoniades and Hunkapiller, 1983), PDGF-AB could be too large to diffuse from the alginate in use while the smaller molecules of TGF-β1 (25 kDA) (Derynck et al., 1986; Roberts et al., 1983) and IGF-1 (7.6 kDA) (Rinderknecht and Humbel, 1978) can escape more easily. The slightly smaller TGF-β1 molecule showed mixed results between the alginate groups of BC and PRP. Perhaps crosslinking between alginate and Ca^2+^ ions found in the blood suspension occurred to a relevant extent in PRP alginate samples, while the higher dilutions in BC samples (Supplementary Material 1) reduced Ca^2+^ levels directly and also indirectly by reducing the concentration of alkaline phosphatase resulting in less crosslinking and lower TGF-β1 retention. An increased release of TGF-β1 after sodium alginate gel treatment of wounds, however, has recently been shown by Choi et al. (2024) and supports the increased concentrations found for BC alginate.

In addition to molecular size, electrostatic effects between negatively charged alginate chains (Tavor Re’em, 2018; Wu et al., 2016) and positive charges of the growth factors investigated in this work have been recognized (Ruvinov et al., 2011) and should be kept in mind as possibly also influencing the respective release patterns. However, a more detailed investigation is required in order to correctly interpret these interactions in this context. Changes in molecular weight distributions of alginate would influence release properties based on the molecular size of the released molecule (Seidenstuecker et al., 2017). Further experiments using alginate with different molecular weight distributions could be carried out to confirm this effect as a driving factor for the observed pattern.

Loading of β-TCP using flow chambers and alginate coating has already been shown for different drugs (Ritschl et al., 2023; Kuehling et al., 2022; Seidenstuecker et al., 2017). The current work first demonstrates a similar release of substances found in human blood suspensions. Consequently, the release of different substances originating from blood, and generally larger molecules than previously considered, can be analyzed in a similar manner. The use of loaded dowels, perhaps in situations where unloaded dowels would have been used anyway, to boost the positive properties of the dowels even further, or in entirely new indications solely to deliver growth factors, might be considered and offer a new form of administration compared to past methods like soaking or simple addition (Szponder et al., 2018; Koksi et al., 2023; Plachokova et al., 2007; Arai et al., 2021; Zhang et al., 2018; Omata et al., 2014).

## 5 Conclusion

We demonstrated the release of different growth factors from β-TCP dowels into the surrounding medium depending on the type of blood suspension used for loading and depending on alginate coating. TGF-β1 and PDGF-AB were released from the ceramics, likely due to active release from platelets found in BC and PRP, with slightly different temporal patterns. IGF-1 release from the ceramics was highest in PPP samples and can mainly be explained by differences in dilution between sample types. Consequentially, observed differences in IGF-1 were attributed to circulating levels rather than release from platelets, and undiluted samples of each type would likely have comparable IGF-1 concentrations. Alginate coating without crosslinking had different effects on growth factors within the surrounding medium, perhaps slightly reducing IGF-1 levels, strongly decreasing PDGF-AB levels, and potentially even increasing TGF-β1 concentrations, even though the effect was only shown for BC samples.

This work shows how flow chamber loading of β-TCP could be used as a delivery system for growth factors with a unique release pattern based on Ca^2+^ activation of platelets and also functions as a demonstration for loading and release of larger molecules than previously analyzed.

## 6 Limitations

A potential burst release of growth factor induced by shear forces was not excluded by additional investigations but rather deemed unlikely on the basis of the low concentrations found at 2 h after loading.

The buffy coats provided by the Institute for Transfusion Medicine and Gene Therapy, University Freiburg, were stored for an unknown duration of up to 24 h at room temperature before being further processed by our group, allowing a timeframe of unregarded, passive growth factor release from platelets not present in both other groups.

## Data Availability

The raw data supporting the conclusions of this article will be made available by the authors, without undue reservation.
