# Supplementary material for: Release kinetics of growth factors loaded into β-TCP ceramics in an in vitro model
Source: Front Bioeng Biotechnol. 2024 Sep 27;12:1441547. doi: 10.3389/fbioe.2024.1441547 (PMC11466813; doi:10.3389/fbioe.2024.1441547)
Supplement: Supplementary file 1 [file Table1.docx]

**Release kinetics of growth factors loaded into β-TCP ceramics in an in vitro model**

**Marco Waldmann^1^, Marc Bohner^2^, Anna Baghnavi^1^, Bianca Riedel^1^, Michael Seidenstuecker ^1*^**

^1^G.E.R.N. Tissue Replacement, Regeneration & Neogenesis, Department of Orthopedics and Trauma Surgery, Medical Center-Albert-Ludwigs-University of Freiburg, Faculty of Medicine, Albert-Ludwigs-University of Freiburg, Hugstetter Straße 55, 79106 Freiburg, Germany

²RMS Foundation, Bischmattstr. 12, Bettlach 2544, Switzerland

*** Correspondence:**Corresponding Author
michael.seidenstuecker@uniklinik-freiburg.de

**Keywords: β-TCP, PRP, Ceramic, Growth factor, TGF-beta, IGF-1, PDGF-AB.**

# Supplement

| **Sample:** | Concentration: platelets/µl before dilution | Dilution [ml] of samples with 0.9% NaCl necessary to standardize for 450.000 platelets/µl | Undiluted with <100.000 platelets/µl as an entry criterion  [ml] | Dilution factor | Dilution (Approx.) | Average dilution factor per Group | Average Dilution per Group (Approx.) |
| --- | --- | --- | --- | --- | --- | --- | --- |
| **PRP 1** | 528.000 | 1.278 PRP1 + 0.222 NaCl |  | 0.852 | 21:25 |  |  |
| **PRP 2** | 449.500 | 1.5 PRP2 undiluted |  | 1 | No Dilution | 0.895 | 9:10 |
| **PRP 3** | 539.000 | 1.25 PRP3 + 0.25 NaCl |  | 0.833 | 21:25 |  |  |
| **BC 1** | 275.250.0 | 0.245 BC1 + 1.255 NaCl |  | 0.163 | 4:25 |  |  |
| **BC 2** | 255.000.0 | 0.265 BC2 + 1.235 NaCl |  | 0.177 | 9:50 | 0.163 | 4:25 |
| **BC 3** | 304.250.0 | 0.222 BC3 + 1.278 NaCl |  | 0.148 | 3:20 |  |  |
| **PPP 1** | 23.050 |  | 1.5 PPP1 undiluted | 1 | No Dilution |  |  |
| **PPP 2** | 37.250 |  | 1.5 PPP2 undiluted | 1 | No Dilution | - | No Dilution |
| **PPP 3** | 9.700 |  | 1.5 PPP3 undiluted | 1 | No Dilution |  |  |

Supplement 1. Initial platelet concentrations for each sample determined using a Casy TT cell counter. Dilutions for the specific volumes used, as factors and additionally as approximated fractions are shown for each individual sample and as an average per group. PPP samples did not require dilution but had <100.000 platelets/µl as a criterion.
